# Comparative analysis of clinical features of SARS-CoV-2 and adenovirus infection among children

**DOI:** 10.1186/s12985-020-01461-4

**Published:** 2020-12-10

**Authors:** Kuanrong Li, Ling Li, Xianfeng Wang, Hui Li, Jun Chen, Lei Liu, Jianbo Shao, Yi Xu, Liya He, Sitang Gong, Huimin Xia, Huiying Liang

**Affiliations:** 1 Clinical Data Center, Guangzhou Women and Children’s Medical Center, Guangzhou Medical University, Jinsui Road, Guangzhou, 510623 Guangdong China; 2 Department of Pediatric, Third People’s Hospital of Shenzhen, Second Affiliated Hospital of Southern University of Science and Technology, Shenzhen, Guangdong China; 3 Department of Pediatric, Wuhan Children’s Hospital, Wuhan, Hubei China; 4 Department of Infectious Disease, Third People’s Hospital of Shenzhen, Shenzhen, Guangdong China; 5 Department of Pediatric, Guangzhou Women and Children’s Medical Center, Guangzhou Medical University, Guangzhou, Guangdong China; 6 Guangdong Provincial Children’s Medical Research Center, Guangzhou Women and Children’s Medical Center, Guangzhou Medical University, Guangzhou, Guangdong China

**Keywords:** COVID-19, SARS-CoV-2, Adenoviruses, Respiratory infections, Children

## Abstract

**Background:**

The new emerging coronavirus disease 2019 (COVID-19) overall shares similar symptoms with other common respiratory viral infections. We aimed in this study to compare COVID-19 and human adenovirus (HAdV) infections in pediatric patients regarding the frequencies of major clinical symptoms and the potential disparities in laboratory and imaging parameters.

**Methods:**

Following a case–control-like design, we built 72 age-matched pediatric COVID-19 and HAdV patient pairs. Their early symptoms and laboratory and imaging characteristics were then retrieved and compared.

**Results:**

Fever and cough were the most common symptoms for both infections but were seen more often in HAdV than in COVID-19 patients (92% vs. 66% and 60% vs. 18%, respectively). Compared with COVID-19 patients, children with HAdV infection had statistically significantly higher values of neutrophil count, neutrophil percentage, activated partial thromboplastin time, prothrombin time, lactate dehydrogenase, C-reactive protein, procalcitonin but lower values of lymphocyte percentage, total bilirubin, potassium and sodium. Thoracic computed tomography also revealed more anomalies in HAdV patients than in COVID-19 patients (95% vs. 67%).

**Conclusions:**

COVID-19 is an overall less symptomatic and less severe infection at admission compared to HAdV respiratory infection in pediatric population.

## Background

In late December 2019, a novel respiratory infectious disease caused by a new strain of coronavirus, namely severe acute respiratory syndrome coronavirus 2 (SARS-CoV-2), made its first appearance in Wuhan, China and spread rapidly around the world [1]. The disease was later on named coronavirus disease 2019 (COVID-19). As of September 27, 2020, a total of 32,730,945 confirmed COVID-19 cases, including 991,224 deaths, have been reported globally [2].

Children appear to be less affected by COVID-19 and only account for 1–5% of diagnosed COVID-19 cases [3]. Fever and cough are the most commonly seen symptoms in children [4, 5]. Compared with adult COVID-19 patients, pediatric patients manifest relatively mild symptoms and rarely develop severe pneumonia [4, 5].

Human adenoviruses (HAdV) are a family of viruses causing 4–10% of respiratory illnesses in children and infants worldwide [6, 7]. Although COVID-19 and HAdV respiratory infection show similar symptoms such as fever and cough [8, 9], detailed comparisons of the two infections with respect to their early clinical characteristics among pediatric patients have not yet been reported.

The aim of this study was to compare the two infections regarding their early clinical, laboratory, and radiological characteristics among pediatric patients.

## Methods

### Study design and participants

From January 23 to February 23, 2020, a total of 106 children (age range: 0–11 years) were diagnosed with COVID-19 and hospitalized in Guangzhou Women and Children’s Medical Center, Third People’s Hospital of Shenzhen, or Wuhan Children’s Hospital. The diagnosis of COVID-19 followed the Protocol for Novel Coronavirus Pneumonia Diagnosis and Treatment issued by the National Health Commission of the People’s Republic of China [10]. All the patients were laboratory confirmed using real-time reverse transcriptase polymerase chain reaction (RT-PCR) assays on nasopharyngeal swab specimens. Laboratory confirmation was also performed on other common respiratory pathogens including influenza-A virus (H1N1, H3N2, H7N9), influenza B virus, respiratory syncytial virus (RSV), parainfluenza virus, adenovirus, SARS coronavirus (SARS-CoV), and MERS coronavirus (MERS-CoV), and any co-infections were precluded from this study.

In the electronic medical database of Guangzhou Women and Children’s Medical Centre, we identified 570 pediatric patients who were hospitalized with laboratory-confirmed adenovirus respiratory infections between November 1, 2016 and March 31, 2020. Please note that patients with laboratory-confirmed co-infections of other respiratory pathogens were also excluded. Following a case–control-like design, we selected an age-matched patient with respiratory HAdV infection for each of the COVID-19 cases, where an eligible match was defined as the absolute difference in age at admission for a patient pair was no greater than 30 days. If a COVID-19 patient had multiple age-matched HAdV patients, priority would be given to the one of the same sex.

### Data collection procedures

Data on demographics and clinical features were collected from electronic medical records. Chest computed tomography (CT) and laboratory findings were collected from the Picture Archiving and Communication Systems and the Laboratory Information System, respectively. Radiologic abnormalities were determined according to descriptions in the clinical charts.

### Statistical analysis

Quantitative measurements were presented as medians and interquartile ranges (IQRs); qualitative measurements were presented as counts and percentages. Abnormally high or low levels of laboratory findings were defined using age-specific or otherwise universal reference ranges (Additional file 1). Because of the pairing design, differences between groups were analyzed by Wilcoxon signed-ranks test (for continuous data) or McNemar’s chi-square test (for binary data), with *p* value less than 0.05 considered statistically significant. All analyses were conducted with R (version 3.4.6; The R Foundation for Statistical Computing, Vienna, Austria).

This study was approved by the ethics committees of participating hospitals. Written informed consent was obtained from parent or guardians of the pediatric COVID-19 patients.

## Results

Among all the pairs, 72 met the matching criterion and therefore 72 age-matched patient pairs of COVID-19 patients and patients with HAdV respiratory infection were formed for analysis. The median age was 2 years (range: 0–11 years) for both groups, and boys accounted for 63% (45) for HAdV and 61% (44) for COVID-19.

**Fig. 1:**
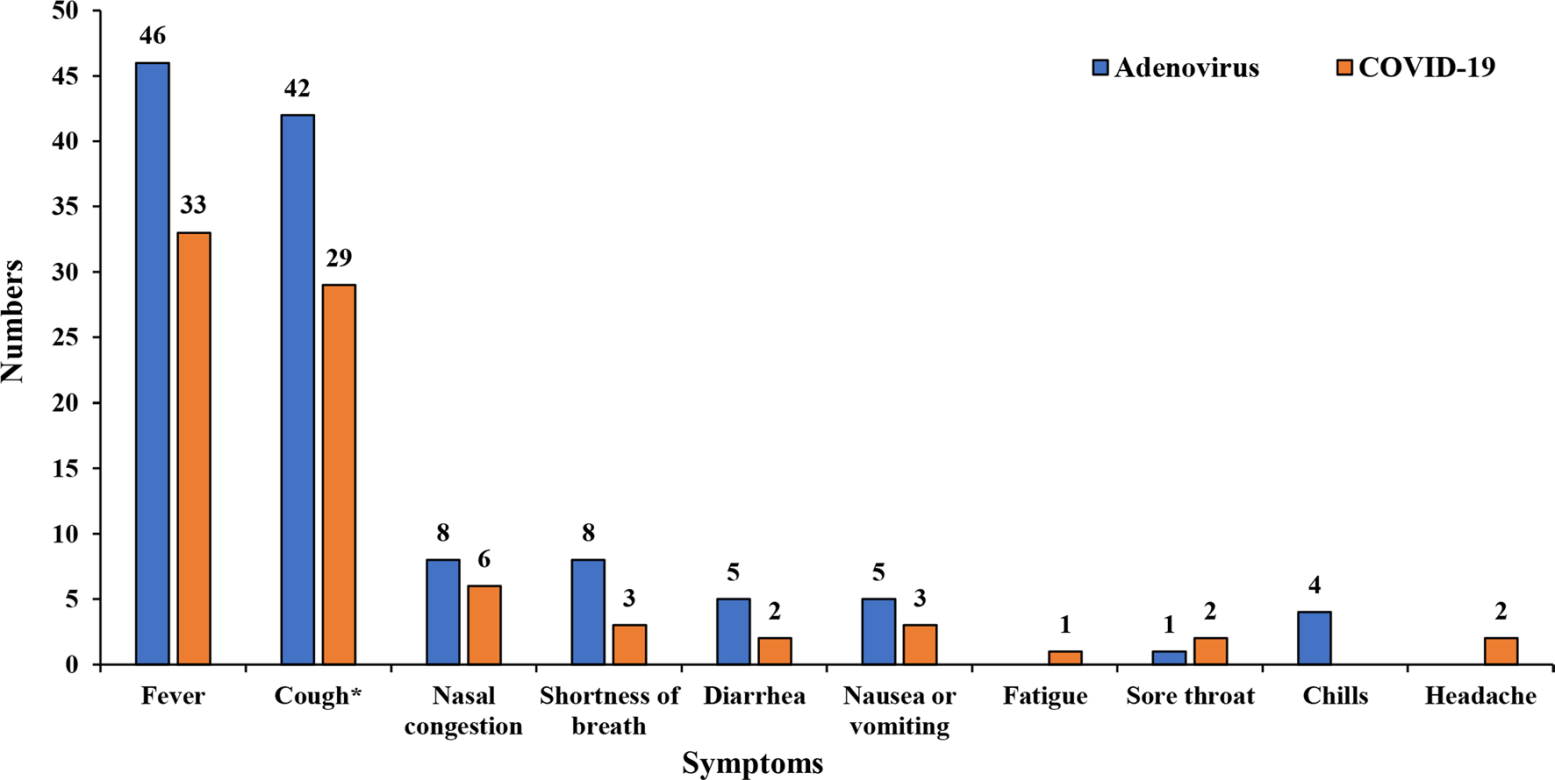
Distributions of symptoms in 50 age-matched pairs of symptomatic pediatric COVID-19 patients and HAdV patients. *Dry cough versus wet cough: 20 versus 9 for COVID-19 and 12 versus 30 for Adenovirus

Twenty-two COVID-19 patients were laboratory confirmed after epidemiological exposure but were clinically asymptomatic at admission. These patients and their HAdV pairs were excluded when we compared the frequencies of clinical symptoms between the two infections. In the remaining 50 symptomatic COVID-19 and HAdV patient pairs, the average time between symptom onset and admission was 3 days for COVID-19 and 7 days for HAdV. The frequencies of the signs and symptoms at admission were reported in Table 1 and Fig. 1. Symptoms at illness onset were similar among the two groups, with fever and cough being the most common symptoms, but the percentages of cases with fever [46 (92%) vs. 33 (66%), *p* = 0.006] and wet cough [30 (60%) vs. 9 (18%), *p* < 0.001] in HAdV were higher than those in COVID-19. More headaches, nasal congestion, shortness of breath, diarrhea, nausea or vomiting, fatigue, and chills were reported in the HAdV group than in the COVID-19 group, but none of these symptoms showed statistically significant difference between the two groups.

**Table 1:** Fever and cough symptoms among 50 age-matched pairs of symptomatic pediatric COVID-19 patients and patients with adenovirus respiratory infection

Sign and symptom	A_yes_C_yes_	A_yes_C_no_	A_no_C_yes_	A_no_C_no_	*p*	OR (95% CI)
Fever	30(60)	16(32)	3(6)	1(2)	0.006	5.33 (1.55–18.33)
Cough	24(48)	18(36)	5(10)	3(6)	0.012	3.60 (1.34–9.70)
Dry cough	6(12)	6(12)	14(28)	24(48)	0.118	0.43 (0.16–1.12)
Wet cough	7(14)	23(46)	2(4)	18(36)	< 0.001	11.50 (2.71–48.78)

Data in the table are count (%) and *OR* (95%*CI*). *p* values were derived from McNemar *χ*^2^ test or exact McNemar *χ*^2^ test. The A and C combinations denote whether a particular symptom was present (subscript yes) or not (subscript no) for an Adenovirus (A) infection and SARS-CoV-2 infection

*CI* confidence interval, *COVID-19* coronavirus disease 19, *OR* odds ratio

Laboratory tests were performed on the day of admission for the HAdV patients and within the first 2 days after admission for the COVID-19 patients. Table 2 listed the laboratory findings showing statistical differences between the two infections. Compared with COVID-19 patients, patients with respiratory HAdV infection had higher levels of neutrophil count [3.8 (2.1–6.7) vs. 2.2 (1.4–3.9), *p* = 0.004], neutrophil percentage [44.5 (35.0–64.3) vs. 35.3 (20.3–48.0), *p* < 0.001], activated partial thromboplastin time (APTT) [43.1 (38.1–47.4) vs. 34.6 (30.9–40.1), *p* < 0.001], prothrombin time (PT) [13.1 (12.5–14.0) vs. 11.4 (10.8–12.7), *p* < 0.001], lactate dehydrogenase (LDH) [342.5 (273.3–484.0) vs. 284.5 (237.3–366.0), *p* = 0.004], C-reactive protein (CRP) [11.7 (3.7–40.2) vs. 0.8 (0.8–5.0), *p* < 0.001] and procalcitonin [0.3 (0.1–1.3) vs. 0.1 (0.0–0.1), *p* < 0.001]. In addition, patients with HAdV infection had averagely lower levels of lymphocyte percentage, total bilirubin, potassium, and sodium than those with SARS-CoV-2 infection. Other laboratory findings did not significantly differ between the two groups (Additional file 2).

**Table 2:** Haematological and blood biochemical measurements of the 72 age-matched pairs of pediatric COVID-19 patients and patients with adenovirus respiratory infection

Characteristics	Adenovirus (N = 72)	COVID-19 (N = 72)	*p*
Blood routine (n = 58)			
Lymphocyte percentage (IQR) (%)	41.5(26.8–53.8)	53.9(40.6–68.9)	< 0.001
Neutrophil count (IQR) (× 10^9^**/**L)	3.8(2.1–6.7)	2.2(1.4–3.9)	0.004
Neutrophil percentage (IQR) ( × 10^9^**/**L)	44.5(35.0–64.3)	35.3(20.3–48.0)	< 0.001
Hemoglobin (IQR) (g/L)	111.0(101.8–121.3)	121.0(113.3–128.5)	< 0.001
Coagulation function (n = 52)			
APTT (IQR) (s)	43.1(38.1–47.4)	34.6(30.9–40.1)	< 0.001
PT (IQR) (s)	13.1(12.5–14.0)	11.4(10.8–12.7)	< 0.001
Blood biochemistry			
Albumin (IQR) (g/L), n = 64	38.0(33.7–41.3)	44.3(42.1–46.4)	< 0.001
Total bilirubin (IQR) (µmol/L), n = 64	3.95(3.02–5.63)	6.25(4.48–9.48)	< 0.001
LDH (IQR) (U/L), n = 64	342.5(273.3–484.0)	284.5(237.3–366.0)	0.004
CRP (IQR) (mg/L), n = 60	11.7(3.7–40.2)	0.8(0.8–5.0)	< 0.001
Procalcitonin (IQR) (ng/L), n = 44	0.3(0.1–1.3)	0.1(0.0–0.1)	< 0.001
Potassium (IQR) (mmol/L), n = 67	3.8(3.4–4.2)	4.5(4.1–5.1)	< 0.001
Sodium (IQR) (mmol/L), n = 67	135.0(132.0–138.3)	138.5(137.0–140.0)	< 0.001

For each measurement, the exact number of patient pairs included in the analysis varied due to missing values. Distribution of the measurements is denoted by median and interquartile range (in parentheses). The *p* values were calculated using Wilcoxon signed-rank test

*APTT* activated partial thromboplastin time, *COVID-19* coronavirus disease 19, *CRP* C-reactive protein, *IQR* interquartile range, *LDH* lactate dehydrogenase, *PT* prothrombin time

As shown in Table 3, the odds of having abnormally low neutrophil count were lower in the HAdV patients than in the COVID-19 patients, but the odds of having higher-than-normal levels of hemoglobin, albumin, APTT, CRP, and procalcitonin were higher in HAdV patients. Data for other laboratory variables showing no statistically significant difference are provided in Additional file 3.

**Table 3:** Haematological and blood biochemical measurements of the 72 age-matched pairs of pediatric COVID-19 patients and patients with adenovirus respiratory infection: abnormally high or low

Characteristics	A_yes_C_yes_	A_yes_C_no_	A_no_C_yes_	A_no_C_no_	*p*	OR (95%CI)
Blood routine (n = 58)						
Neutrophils counts						
Abnormally low	7(12)	6(10)	17(29)	28(48)	0.037	0.35(0.14–0.90)
Hemoglobin						
Abnormally low	3(5)	16(28)	3(5)	36(62)	0.006	5.33(1.55–18.30)
Coagulation function (n = 52)						
APTT						
Abnormally high	1(2)	16(31)	1(2)	34(65)	< 0.001	16.00(2.12–120.65)
Blood biochemistry						
Albumin (n = 64)						
Abnormally low	8(13)	35(55)	1(2)	20(31)	< 0.001	35.00(4.79–255.48)
CRP (n = 60)						
Abnormally high	8(13)	31(52)	2(3)	19(32)	< 0.001	15.50(3.71–64.80)
Procalcitonin(n = 44)						
Abnormally high	11(25)	23(52)	1(2)	9(21)	< 0.001	23.00(3.11–170.32)
Sodium (n = 67)						
Abnormally low	17(25)	28(42)	9(13)	13(19)	0.003	3.11(1.47–6.59)

For each measurement, the exact number of patient pairs included into the analysis varied due to missing values. Data are presented in count and percentage (in parentheses). The *p* values were calculated using McNemar's *χ*^2^ test. The A and C combinations denote whether a particular measurement was abnormally high/low (subscript yes) or not (subscript no) for an Adenovirus (A) and COVID-19 (C) patient pair

*APTT* activated partial thromboplastin time, *CI* confidence interval, *COVID-19* coronavirus disease 19, *CRP* C-reactive protein, *OR* odds ratio

The CT investigations were performed within the first 2 days after admission for COVID-19 patients. However, only 21 HAdV children had chest CT examinations upon admission, and therefore we compared the CT findings only in 21 patient pairs. CT abnormality was found in 14 (67%) patients with COVID-19 and 20 (95%) patients with respiratory HAdV infection. There was no difference in the frequencies of ground-glass opacity [4 (19%) vs. 4 (19%)] and unilateral patchy shadowing [6 (29%) vs. 5 (24%)] between the two groups, but bilateral patchy shadowing in HAdV was more often seen in COVID-19 patients (52.4% vs. 14.3%, *p* = 0.027).

## Discussion

In this study, we presented the early clinical symptoms and signs, radiologic and laboratory findings of of pediatric patients who had HAdV respiratory infection and COVID-19, respectively. Fever, cough, nasal congestion, shortness of breath, diarrhea, nausea or vomiting, and chills were the most often reported early symptoms in both infections but occurred more often in patients with HAdV infection. HAdV patients had higher levels of neutrophil count, neutrophil percentage, LDH, CRP, procalcitonin, and APTT, but lower levels of lymphocyte percentage and total bilirubin. CT abnormality, in particular bilateral patchy shadowing, occurred more often in HAdV patients than in COVID-19 patients.

Children with COVID-19 often appear to have a mild clinical course [11–13]. In the present study, only 1 case was treated in ICU, and no one died during hospitalization. In a previous report, asymptomatic COVID-19 patients were common at admission as patients with a history of household exposure or travel to epidemic areas were required to undergo free testing, isolation, or hospitalization [14], while children with respiratory HAdV infection were often admitted because of certain symptoms. Thus, we only retained symptomatic COVID-19 and HAdV patient pairs for the symptomological comparison. Our findings were consistent with prior research [11, 13, 15, 16] where fever and cough were found to be the most common clinical manifestations. We found that children with HAdV infection were more likely to exhibit clinical symptoms than COVID-19 cases. Previous research assumed that low frequencies of clinical symptoms in pediatric COVID-19 patients might relate to children’s less mature and functional binding receptors of the target cells [11]. It may also be because the COVID-19 cases in the present study had an averagely shorter time between symptom onset and hospital admission than patients with HAdV infection.

Previous studies showed that elevated neutrophils and decreased lymphocytes signify adverse clinical progress and an increased risk of poor prognosis in both SARS-CoV-2 and HAdV infections [17–19]. In this study, children with HAdV infection showed a higher neutrophil count and a lower level of lymphocyte percentage than COVID-19 patients, indicating that two infections might be different in their severity. Previous literature showed that longer APTT and PT were related to poor outcome in COVID-19 [20]. In this study, markedly longer APTT and PT were seen in children with HAdV infection, implying that pediatric HAdV patients might have more severe coagulation disorder than COVID-19 patients. With respect to immunologic biomarkers, significantly higher levels of C-reactive protein and procalcitonin were observed for children with HAdV infection. The elevation of these two markers points to the development of a systemic inflammatory response syndrome (SIRS) in patients with a severe form of respiratory disease [17, 21, 22].

In the present study, other hematological tests than what we reported above showed statistically non-significant differences in their levels between the COVID-19 patients and patients with HAdV infection. However, some of these tests might also be indicators of COVID-19 severity. A recent meta-analysis reports severe COVID-19 associated with lower lymphocyte and higher leukocyte counts, although only adult patients were considered in the analysis [10]. Increased serum creatinine was also reported in pediatric patients as a marker of acute kidney injury [23]. However, none of these hematological tests in the present study showed statistically significant differences in their levels between the COVID-19 patients and patients with HAdV infection.

CT investigations in pediatric COVID-19 patients are limited but consistently show that bilateral involvement occur more often than unilateral involvement and ground-glass opacity is the most common CT abnormalities [24]. In this study, the rate of bilateral patchy shadowing was also higher than the rate of unilateral patchy shadowing but only 19% of our COVID-19 cases showed ground-glass opacity, possibly due to the fact that CT investigation was performed at the very early stage of the disease.

This study, to the best of our knowledge, is the first study that compares the SARS-CoV-2 and HAdV infection in the pediatric population. Considering its small sample size and inclusion mainly of patients with mild clinical manifestations, future studies should focus on more representative pediatric COVID-19 patients and identification among them of clinical features that can help distinguish this infection from other common respiratory viral infections in their early stage.

## Conclusions

COVID-19 is an overall less symptomatic and less severe infection at admission compared to HAdV respiratory infection in pediatric population.

## Supplementary Information

**Additional file 1: Table S1.** Reference ranges for haematological and blood biochemical measurements used in this study. The reference ranges for leucocyte count, platelet count, lymphocyte percentage, and neutrophil percentage are age-specific and those for the others are universal for all age groups.

**Additional file 2: Table S2.** Other haematological and blood biochemical measurements of the 72 age-matched pairs of pediatric COVID-19 patients and patients with adenovirus respiratory infection. For each measurement, the exact number of patient pairs included in the analysis varied due to missing values. Distribution of the measurements is denoted by median and interquartile range (in parentheses). The P values were calculated using Wilcoxon signed-rank test. ALT: alanine aminotransferase, AST: aspartate aminotransferase, BUN: blood urea nitrogen, CK: creatine kinase, COVID-19: coronavirus disease 19, IQR: interquartile range.

**Additional file 3: Table S3.** Other haematological and blood biochemical measurements of the 72 age-matched pairs of pediatric COVID-19 patients and patients with adenovirus respiratory infection: abnormally high or low. For each measurement, the exact number of patient pairs included into the analysis varied due to missing values. Data are presented in count and percentage (in parentheses). The P values were calculated using McNemar χ^2^ test. The A and C combinations denote whether a particular measurement was abnormally high/low (subscript yes) or not (subscript no) for an Adenovirus (A) and COVID-19 (C) patient pair. †OR was not calculable due to the zero value for the AnoCyes combination. ALT: alanine aminotransferase, AST: aspartate aminotransferase, BUN: blood urea nitrogen, CI: Confidence interval, CK: creatine kinase, COVID-19: coronavirus disease 19, LDH: lactate dehydrogenase, OR: odds ratio, PT: prothrombin time.

## Data Availability

The datasets analyzed during the current study are not publicly available but are available from the corresponding author on reasonable request.

## Abbreviations

SARS-CoV-2: Severe acute respiratory syndrome coronavirus 2
COVID-19: Coronavirus disease 2019
HAdV: Human adenoviruses
RT-PCR: Real-time reverse transcription polymerase chain reaction
CT: Computed tomography
APTT: Activated partial thromboplastin time
PT: Prothrombin time
LDH: Lactate dehydrogenase
CRP: C-reactive protein
ICU: Intensive care unit
SIRS: Systemic inflammatory response syndrome
OR: Odds ratio
IQR: Interquartile range
CI: Confidence interval
