# Supplementary material for: Comparative analysis of clinical features of SARS-CoV-2 and adenovirus infection among children
Source: Virol J. 2020 Dec 10;17:193. doi: 10.1186/s12985-020-01461-4 (PMC7726278; doi:10.1186/s12985-020-01461-4)
Supplement: Supplementary file 1 — Additional file 1: Table S1. Reference ranges for haematological and blood biochemical measurements used in this study. The reference ranges for leucocyte count, platelet count, lymphocyte percentage, and neutrophil percentage are age-specific and those for the others are universal for all age groups. [file 12985_2020_1461_MOESM1_ESM.docx]

| **Table S1. Reference ranges for haematological and blood biochemical measurements used in this study*** | | | | | |
| --- | --- | --- | --- | --- | --- |
| **Laboratory findings** | **0 ~14 d** | **15 ~30 d** | **31d~<0.5 y** | **0.5 ~<6 y** | **6 ~<18 y** |
| Leucocyte count (× 10⁹/L) | 4.94-27.48 | 7.80-15.91 | 6.00-14.99 | 4.86-13.51 | 3.84-11.4 |
| Platelet count (× 10⁹/L) | 144-450 | 248-586 | 229-597 | 189-459 | 175-369 |
| Lymphocyte percentage (%) | 24.9-68.5 | 31.9-82.7 | 30.4-86.7 | 18.1-79.9 | 15.5-57.8 |
| Neutrophil percentage (%) | 15.2-66.1 | 10.6-57.3 | 8.9-76.0 | 16.9-74.0 | 28.6-74.7 |
| Lymphocyte count (× 10⁹/L) | 1.55-4.80 | | | | |
| Neutrophil count (× 10⁹/L) | 2.0-7.2 | | | | |
| Hemoglobin level (g/L) | 105-145 | | | | |
| Activated partial thromboplastin time (s) | 28-45 | | | | |
| Prothrombin time (s) | 11-15 | | | | |
| Albumin level (g/L) | 40–55 | | | | |
| Alanine aminotransferase level (U/L) | 9–50 | | | | |
| Aspartate aminotransferase level (U/L) | 5-60 | | | | |
| Total bilirubin level (μmol/L) | 2–17 | | | | |
| Blood urea nitrogen level (mmol/L) | 2.1-7.1 | | | | |
| Serum creatinine level (μmol/L) | 18-62 | | | | |
| Creatine kinase level (U/L) | 45-390 | | | | |
| Lactate dehydrogenase level (U/L) | 159-322 | | | | |
| Glucose level (mmol/L) | 4.1-5.9 | | | | |
| C-reactive protein level (mg/L) | 0-6 | | | | |
| Procalcitonin level (ng/mL) | 0-0.1 | | | | |
| Potassium level (mmol/L) | 3.4-5.7 | | | | |
| Sodium level (mmol/L) | 138-144 | | | | |

*The reference ranges for leucocyte count, platelet count, lymphocyte percentage, and neutrophil percentage are age-specific and those for the others are universal for all age groups.
