# Supplementary material for: Comparative analysis of clinical features of SARS-CoV-2 and adenovirus infection among children
Source: Virol J. 2020 Dec 10;17:193. doi: 10.1186/s12985-020-01461-4 (PMC7726278; doi:10.1186/s12985-020-01461-4)
Supplement: Supplementary file 2 — Additional file 2: Table S2. Other haematological and blood biochemical measurements of the 72 age-matched pairs of pediatric COVID-19 patients and patients with adenovirus respiratory infection. For each measurement, the exact number of patient pairs included in the analysis varied due to missing values. Distribution of the measurements is denoted by median and interquartile range (in parentheses). The P values were calculated using Wilcoxon signed-rank test. ALT: alanine aminotransferase, AST: aspartate aminotransferase, BUN: blood urea nitrogen, CK: creatine kinase, COVID-19: coronavirus disease 19, IQR: interquartile range. [file 12985_2020_1461_MOESM2_ESM.docx]

| **Table S2. Other haematological and blood biochemical measurements of the 72 age-matched pairs of pediatric COVID-19 patients and patients with adenovirus respiratory infection *** | | | |
| --- | --- | --- | --- |
| **Characteristics** | **Adenovirus(N=72)** | **COVID-19(N=72)** | ***P*** |
| **Blood routine(n=58)** |  |  |  |
| Leucocyte count (IQR), × 10⁹/L | 9.3(5.2-12.4) | 7.1(5.5-10.4) | 0.134 |
| Lymphocyte count (IQR), × 10⁹/L | 3.3(1.5-5.2) | 3.6(2.4-5.1) | 0.143 |
| Platelet count (IQR), × 10⁹/L | 278.0(217.5-400.8) | 287.0(220.8-380.8) | 0.792 |
| **Blood biochemistry** |  |  |  |
| ALT (IQR), U/L, n=57 | 16.0(11.0-23.0) | 20.0(12.0-28.5) | 0.194 |
| AST (IQR), U/L, n=57 | 40.0(30.0-53.0) | 39.9(32.5-51.1) | 0.321 |
| BUN (IQR), mmol/L, n=39 | 3.2(2.3-4.0) | 3.9(2.5-4.7) | 0.051 |
| Serum creatinine (IQR), μmol/L, n=64 | 25.0(19.0-30.8) | 26.0(21.0-29.9) | 0.185 |
| CK (IQR), U/L, n=62 | 92.5(67.0-185.5) | 98.7(79.8-153.5) | 0.864 |
| Glucose (IQR), mmol/L, n=25 | 5.7(4.9-7.2) | 5.4(4.8-6.0) | 0.568 |
| *For each measurement, the exact number of patient pairs included in the analysis varied due to missing values. Distribution of the measurements is denoted by median and interquartile range (in parentheses). The *P* values were calculated using Wilcoxon signed-rank test. ALT: alanine aminotransferase, AST: aspartate aminotransferase, BUN: blood urea nitrogen, CK: creatine kinase, COVID-19: coronavirus disease 19, IQR: interquartile range. | | | |
