# Supplementary material for: Comparative analysis of clinical features of SARS-CoV-2 and adenovirus infection among children
Source: Virol J. 2020 Dec 10;17:193. doi: 10.1186/s12985-020-01461-4 (PMC7726278; doi:10.1186/s12985-020-01461-4)
Supplement: Supplementary file 3 — Additional file 3: Table S3. Other haematological and blood biochemical measurements of the 72 age-matched pairs of pediatric COVID-19 patients and patients with adenovirus respiratory infection: abnormally high or low. For each measurement, the exact number of patient pairs included into the analysis varied due to missing values. Data are presented in count and percentage (in parentheses). The P values were calculated using McNemar χ2 test. The A and C combinations denote whether a particular measurement was abnormally high/low (subscript yes) or not (subscript no) for an Adenovirus (A) and COVID-19 (C) patient pair. †OR was not calculable due to the zero value for the AnoCyes combination. ALT: alanine aminotransferase, AST: aspartate aminotransferase, BUN: blood urea nitrogen, CI: Confidence interval, CK: creatine kinase, COVID-19: coronavirus disease 19, LDH: lactate dehydrogenase, OR: odds ratio, PT: prothrombin time. [file 12985_2020_1461_MOESM3_ESM.docx]

| **Table S3. Other haematological and blood biochemical measurements of the 72 age-matched pairs of pediatric COVID-19 patients and patients with adenovirus respiratory infection: abnormally high or low*** | | | | | | |
| --- | --- | --- | --- | --- | --- | --- |
| **Characteristics** | **A_yes_C_yes_** | **A_yes_C_no_** | **A_no_C_yes_** | **A_no_C_no_** | ***P*** | **OR (95%CI)** |
| **Blood routine (n=58)** |  |  |  |  |  |  |
| Leucocyte count |  |  |  |  |  |  |
| Abnormally low | 0(0) | 5(9) | 8(14) | 45(78) | 0.579 | 0.63(0.20, 1.91) |
| Abnormally high | 1(2) | 6(10) | 4(7) | 47(81) | 0.752 | 1.50(0.42, 5.31) |
| Lymphocyte count |  |  |  |  |  |  |
| Abnormally low | 3(5) | 11(19) | 4(7) | 40(69) | 0.121 | 2.75(0.88, 8.64) |
| Abnormally high | 10(17) | 7(12) | 8(14) | 33(57) | 1.000 | 0.88(0.31, 2.50) |
| Lymphocyte percentage |  |  |  |  |  |  |
| Abnormally low | 0(0) | 9(16) | 4(7) | 45(78) | 0.267 | 2.25(0.69, 7.31) |
| Abnormally high | 1(2) | 0(0) | 5(9) | 52(90) | 0.063 | 0.00(0.00, 1.09) |
| Neutrophil count |  |  |  |  |  |  |
| Abnormally high | 1(2) | 11(19) | 6(10) | 40(69) | 0.333 | 1.83(0.68, 4.96) |
| Neutrophil percentage |  |  |  |  |  |  |
| Abnormally low | 0(0) | 3(5) | 6(10) | 49(85) | 0.508 | 0.50(0.08, 2.34) |
| Abnormally high | 0(0) | 6(10) | 2(3) | 50(86) | 0.289 | 3.00(0.54, 30.39) |
| Platelet count |  |  |  |  |  |  |
| Abnormally low | 0(0) | 4(7) | 10(17) | 44(76) | 0.181 | 0.40(0.12, 1.28) |
| Abnormally high | 0(0) | 3(5) | 5(9) | 50(86) | 0.727 | 0.60(0.09, 3.08) |
| Haemoglobin |  |  |  |  |  |  |
| Abnormally high | 1(2) | 2(3) | 2(3) | 53(91) | 1.000 | 1.00(0.07, 13.80) |
| **Coagulation function (n=52)** |  |  |  |  |  |  |
| PT† |  |  |  |  |  |  |
| Abnormally high | 0(0) | 3(6) | 0(0) | 49(94) | 0.250 | — |
| **Blood biochemistry** |  |  |  |  |  |  |
| ALT (n=57) |  |  |  |  |  |  |
| Abnormally high | 0(0) | 4(7) | 3(5) | 50(88) | 1.000 | 1.33(0.23, 9.10) |
| AST (n=57) |  |  |  |  |  |  |
| Abnormally high | 3(5) | 7(12) | 4(7) | 43(75) | 0.547 | 1.75(0.51, 5.98) |
| Total bilirubin (n=64) |  |  |  |  |  |  |
| Abnormally high | 2(3) | 1(2) | 1(2) | 60(94) | 1.000 | 1.00(0.01,78.50) |
| LDH (n=64) |  |  |  |  |  |  |
| Abnormally high | 16(25) | 20(31) | 11(17) | 17(27) | 0.151 | 1.82(0.87, 3.79) |
| BUN (n=39) |  |  |  |  |  |  |
| Abnormally high | 0(0) | 1(3) | 2(5) | 36(92) | 1.000 | 0.50(0.01, 9.60) |
| Serum creatinine (n=64) |  |  |  |  |  |  |
| Abnormally high | 0(0) | 1(2) | 2(3) | 61(95) | 1.000 | 0.50(0.01, 9.60) |
| CK (n=62) |  |  |  |  |  |  |
| Abnormally high | 0(0) | 6(10) | 2(3) | 54(87) | 0.289 | 3.00(0.54, 30.39) |
| Glucose (n=25) |  |  |  |  |  |  |
| Abnormally high | 4(16) | 7(28) | 2(8) | 12(48) | 0.180 | 3.50(0.67, 34.53) |
| Potassium (n=67) |  |  |  |  |  |  |
| Abnormally high | 0(0) | 0(0) | 6(9) | 61(91) | 0.013 | 0.00(0.00-0.85) |
| *For each measurement, the exact number of patient pairs included into the analysis varied due to missing values. Data are presented in count and percentage (in parentheses). The *P* values were calculated using McNemar *χ*² test. The A and C combinations denote whether a particular measurement was abnormally high/low (subscript yes) or not (subscript no) for an Adenovirus (A) and COVID-19 (C) patient pair. †OR was not calculable due to the zero value for the AnoCyes combination. ALT: alanine aminotransferase, AST: aspartate aminotransferase, BUN: blood urea nitrogen, CI: Confidence interval, CK: creatine kinase, COVID-19: coronavirus disease 19, LDH: lactate dehydrogenase, OR: odds ratio, PT: prothrombin time. | | | | | | |
